# Supplementary figures and images for: Clonal landscape of autoantibody-secreting plasmablasts in COVID-19 patients
Source: Life Sci Alliance. 2024 Sep 17;7(12):e202402774. doi: 10.26508/lsa.202402774 (PMC11408605; doi:10.26508/lsa.202402774)

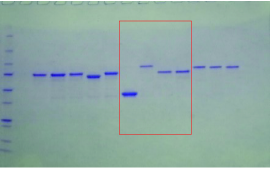

Supplement: Supplementary file 3 [file LSA-2024-02774_SdataFS2.zip › SourceDataForFigureS2/SourceDataForFigure2SD/SourceDataForFigureS2D_gel.tif]

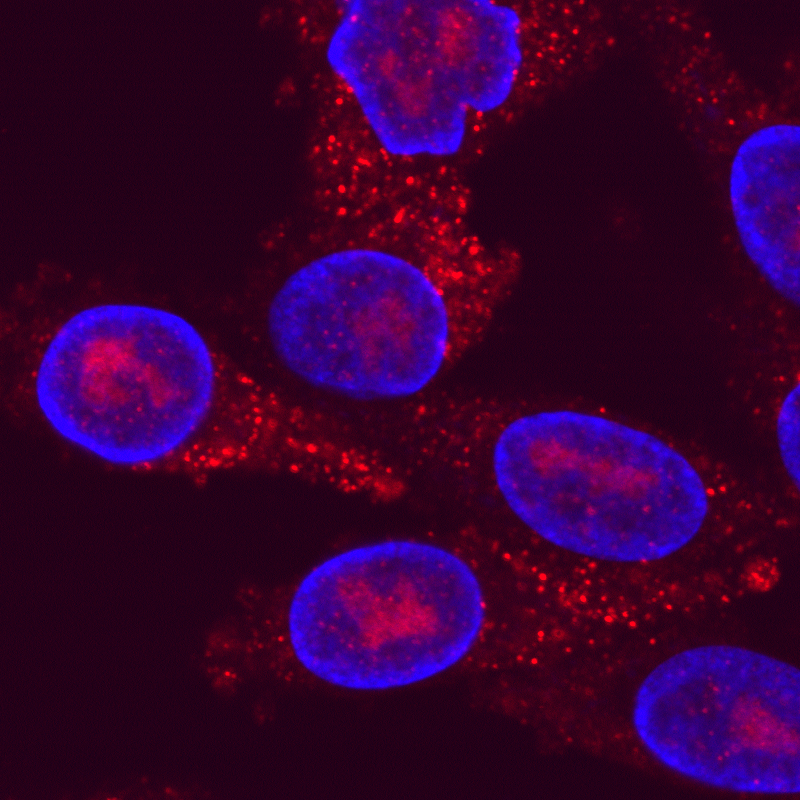

Supplement: Supplementary file 15 [file LSA-2024-02774_SdataF2.4.zip › SourceDataForFigure2D/1_CoV1804.png]

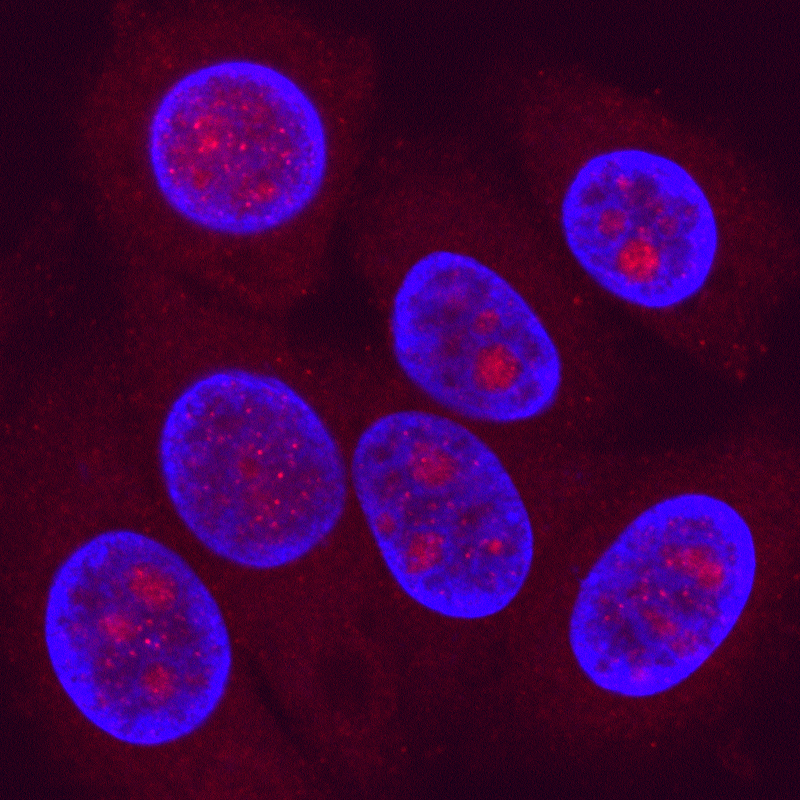

Supplement: Supplementary file 15 [file LSA-2024-02774_SdataF2.4.zip › SourceDataForFigure2D/2_CoV1838.png]

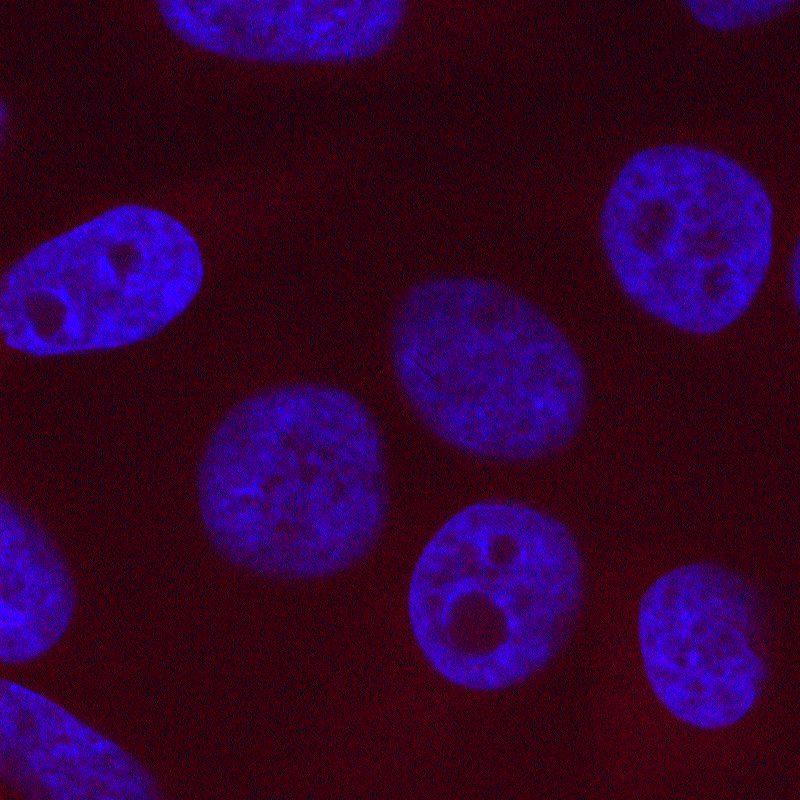

Supplement: Supplementary file 15 [file LSA-2024-02774_SdataF2.4.zip › SourceDataForFigure2D/3_Negative_control.png]

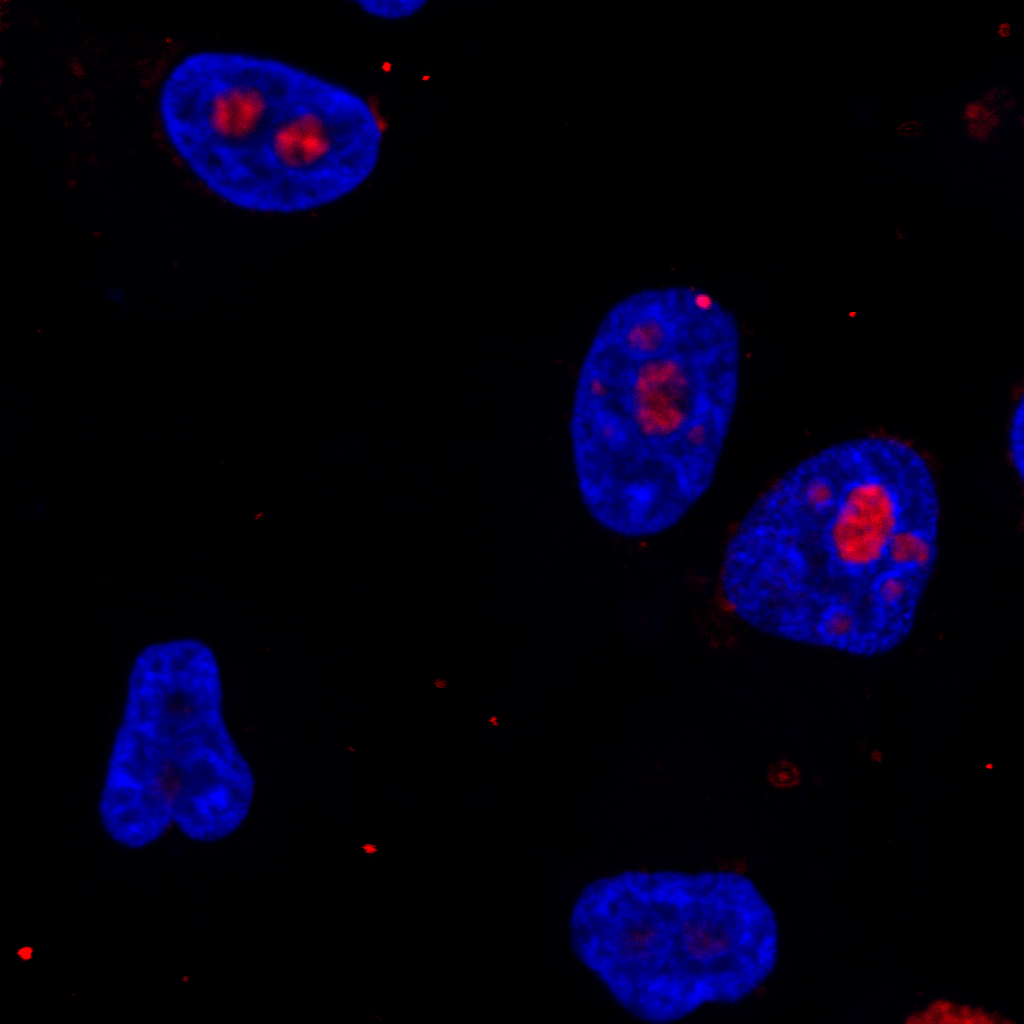

Supplement: Supplementary file 16 [file LSA-2024-02774_SdataF2.5.zip › SourceDataForFigure2E/No119.png]

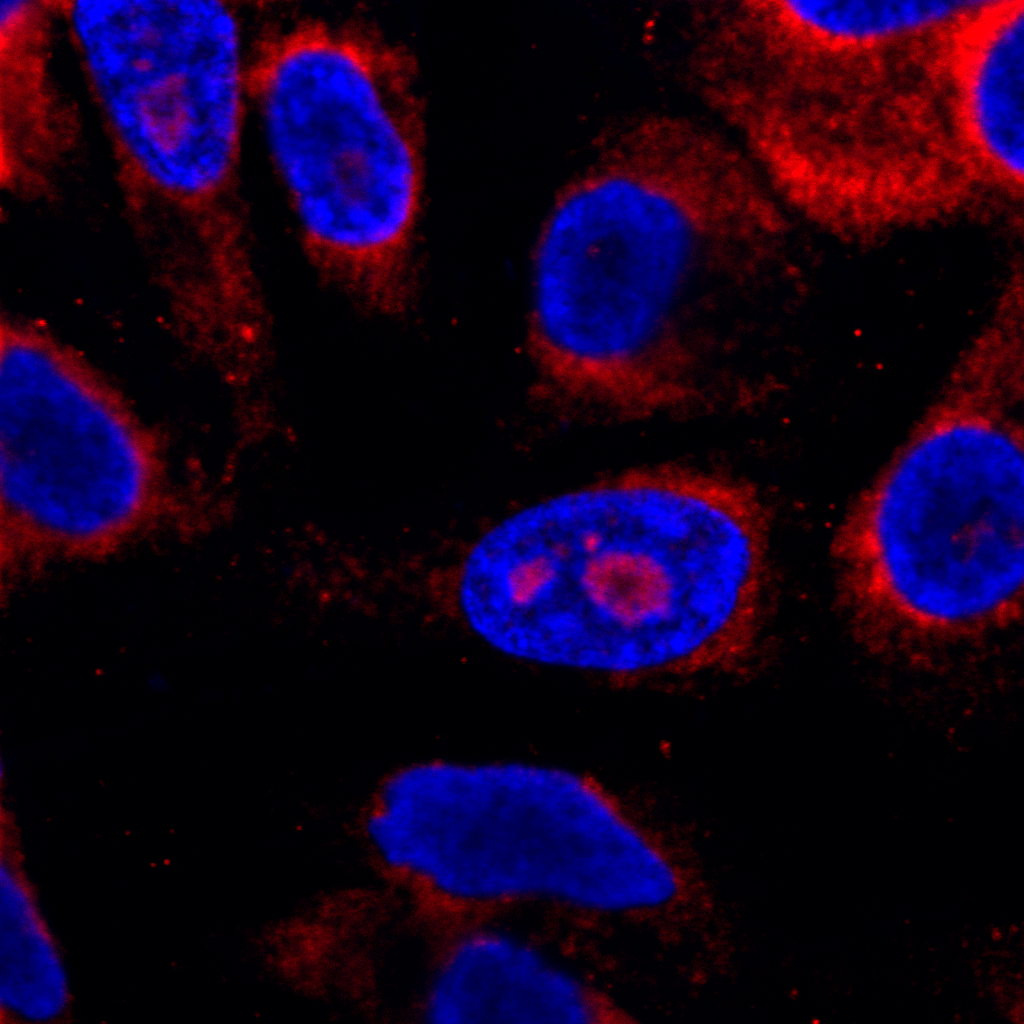

Supplement: Supplementary file 16 [file LSA-2024-02774_SdataF2.5.zip › SourceDataForFigure2E/No91.png]

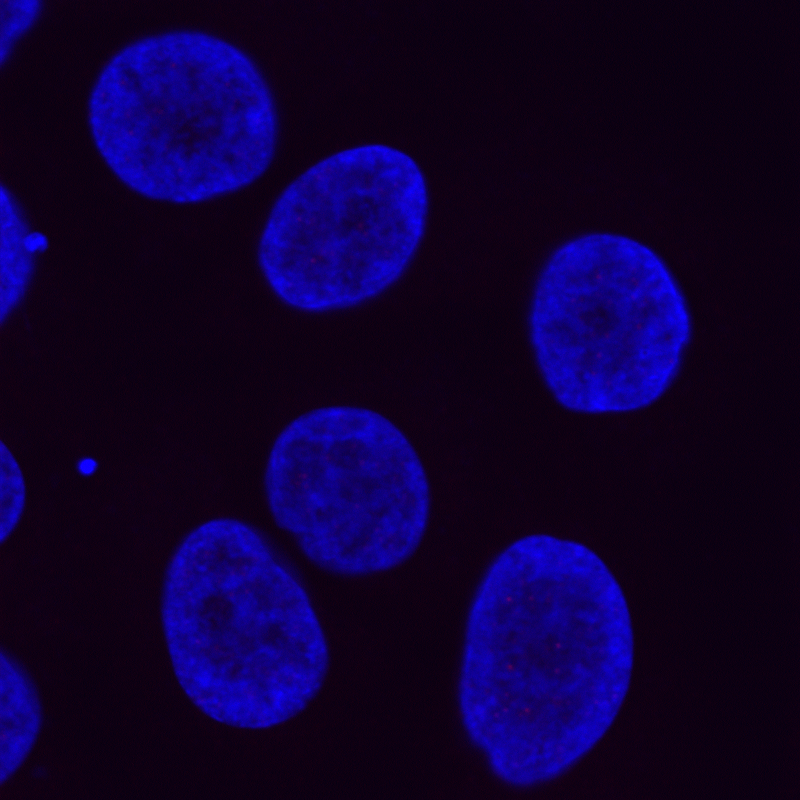

Supplement: Supplementary file 25 [file LSA-2024-02774_SdataF3.3.zip › SourceDataForFigure3E/CoV43678.png]

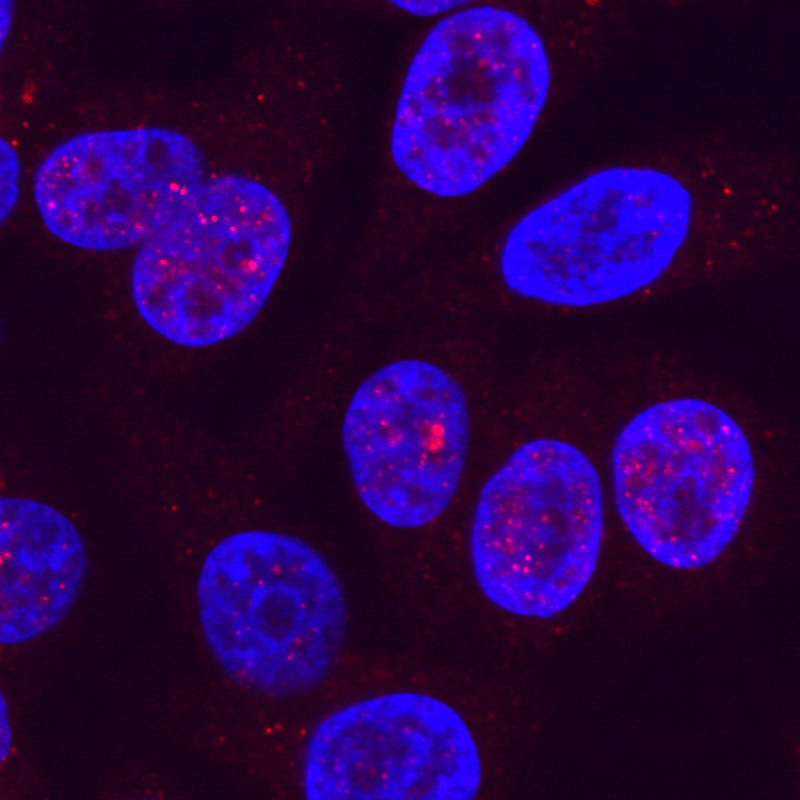

Supplement: Supplementary file 25 [file LSA-2024-02774_SdataF3.3.zip › SourceDataForFigure3E/CoV4803.png]

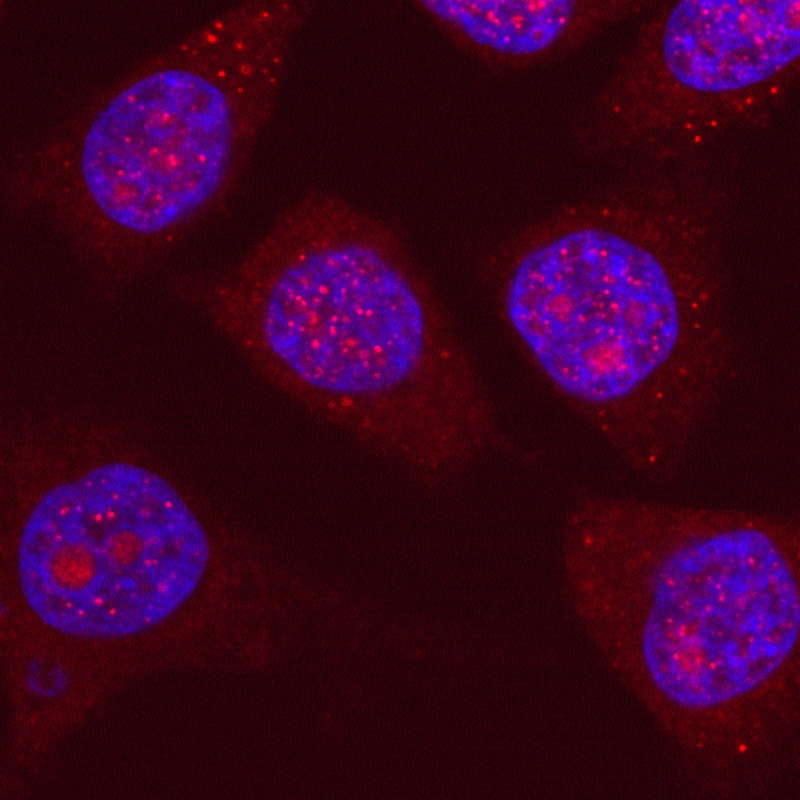

Supplement: Supplementary file 25 [file LSA-2024-02774_SdataF3.3.zip › SourceDataForFigure3E/CoV4842.png]

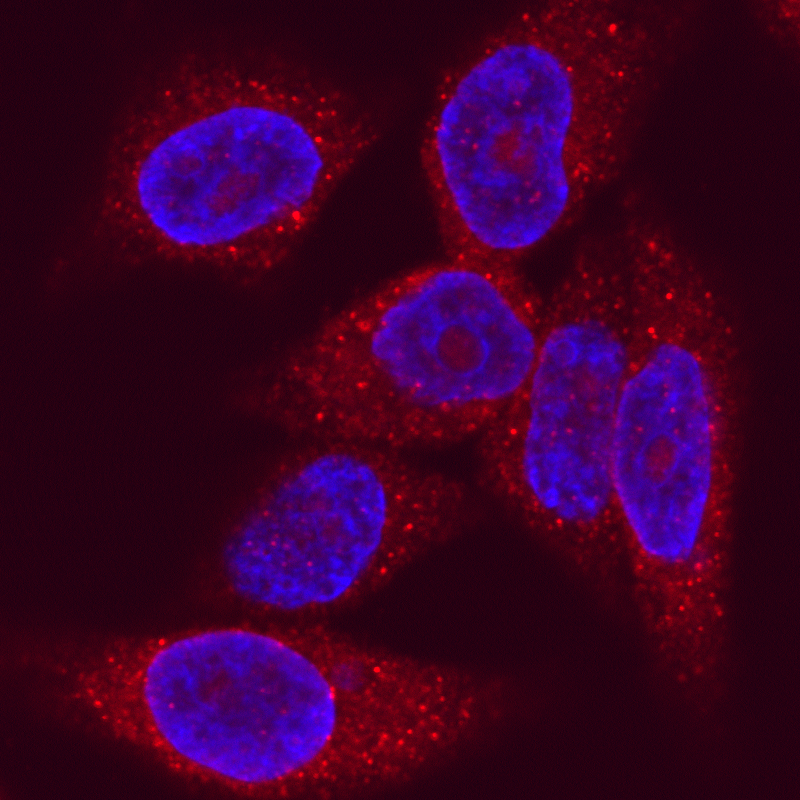

Supplement: Supplementary file 25 [file LSA-2024-02774_SdataF3.3.zip › SourceDataForFigure3E/CoV59020.png]

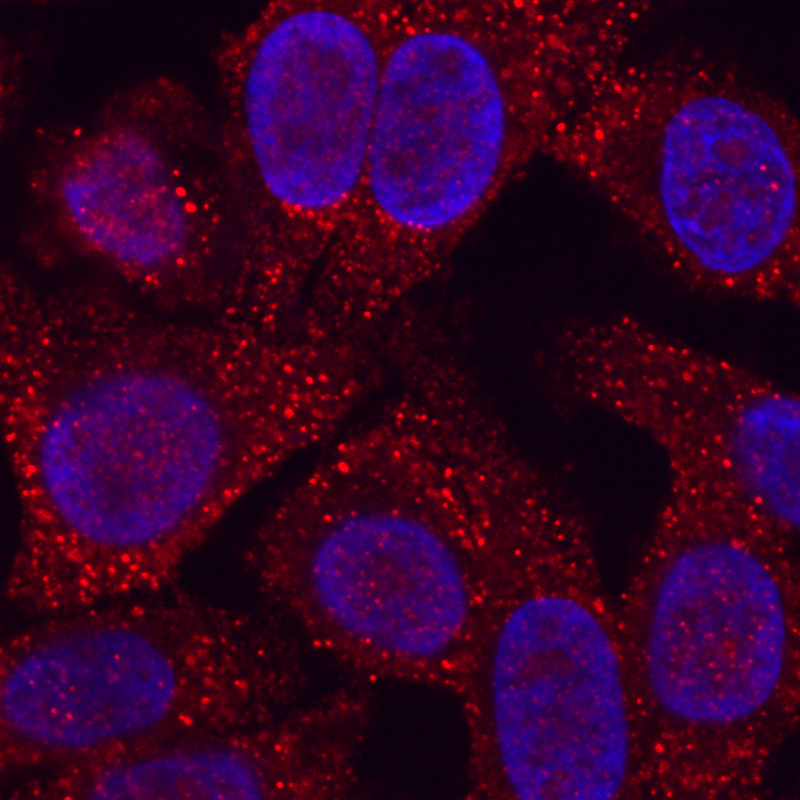

Supplement: Supplementary file 25 [file LSA-2024-02774_SdataF3.3.zip › SourceDataForFigure3E/CoV59030.png]

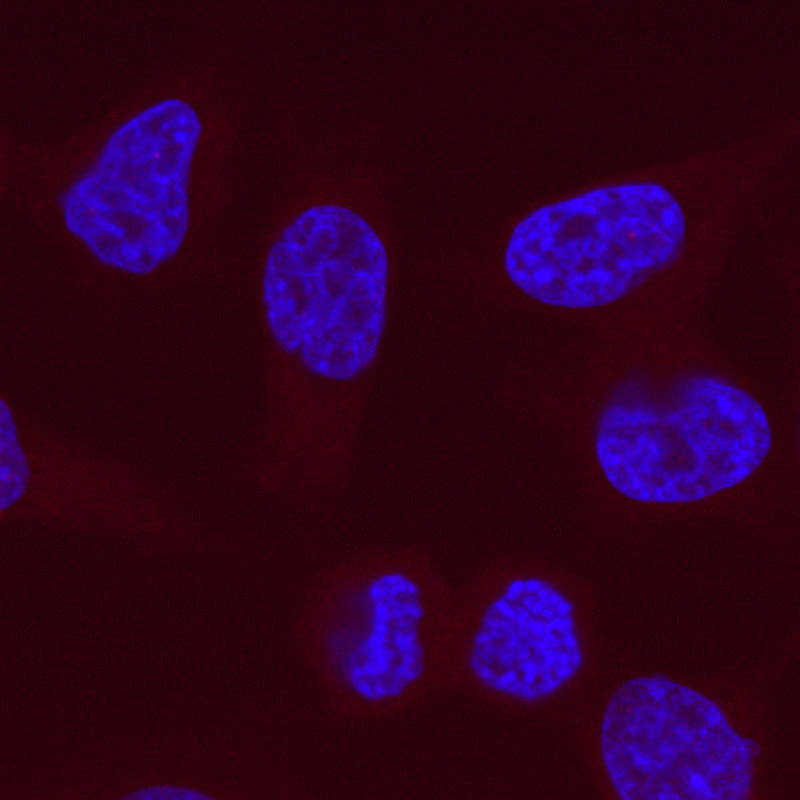

Supplement: Supplementary file 25 [file LSA-2024-02774_SdataF3.3.zip › SourceDataForFigure3E/CoV6725.png]

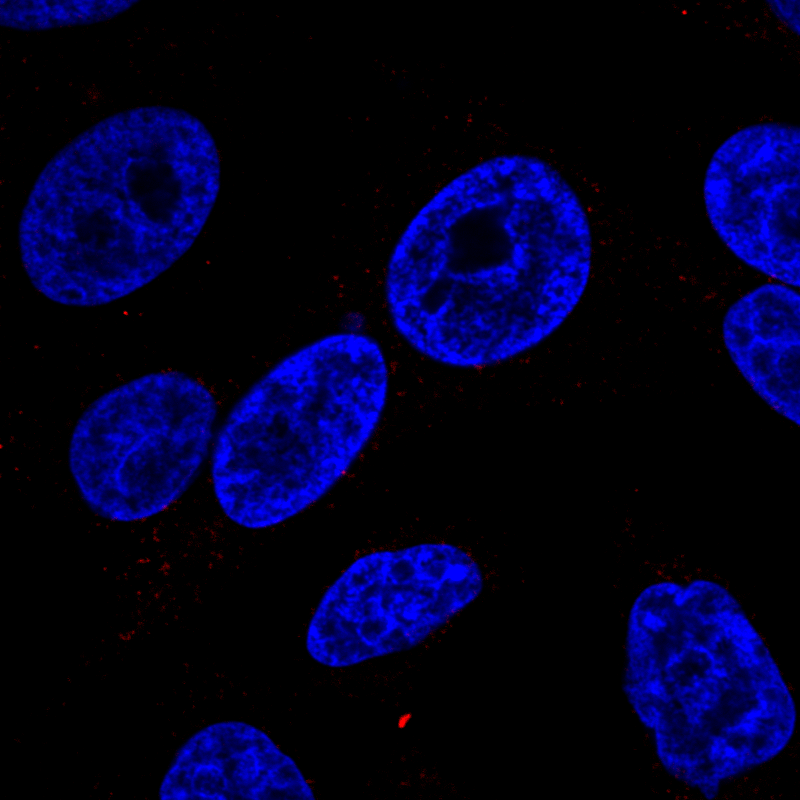

Supplement: Supplementary file 25 [file LSA-2024-02774_SdataF3.3.zip › SourceDataForFigure3E/CoV7699.png]

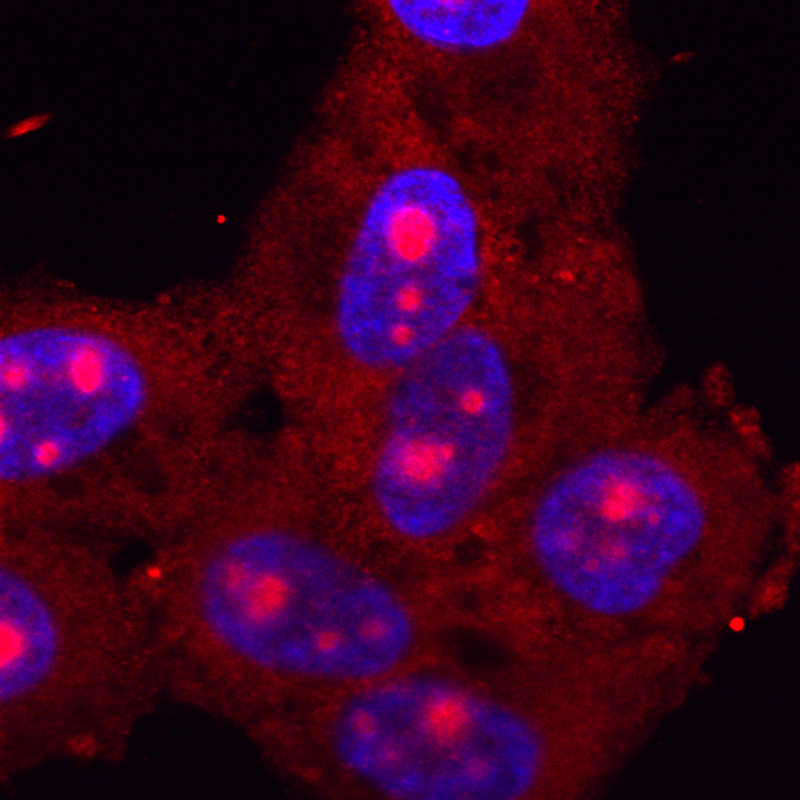

Supplement: Supplementary file 33 [file LSA-2024-02774_SdataF5.2.zip › SourceDataForFigure5B/CoV1804.png]

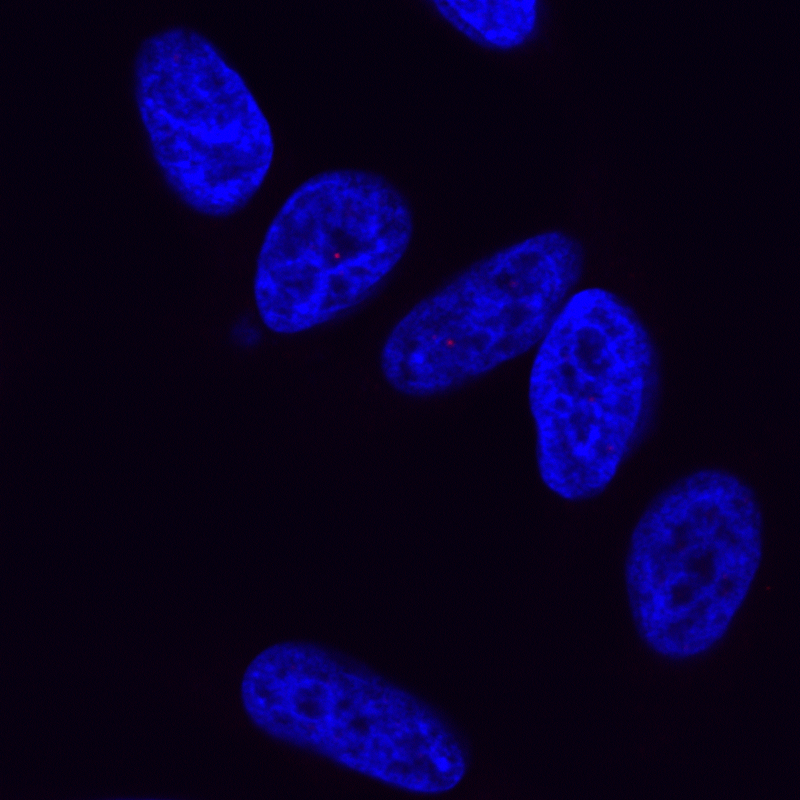

Supplement: Supplementary file 33 [file LSA-2024-02774_SdataF5.2.zip › SourceDataForFigure5B/CoV1804GL.png]

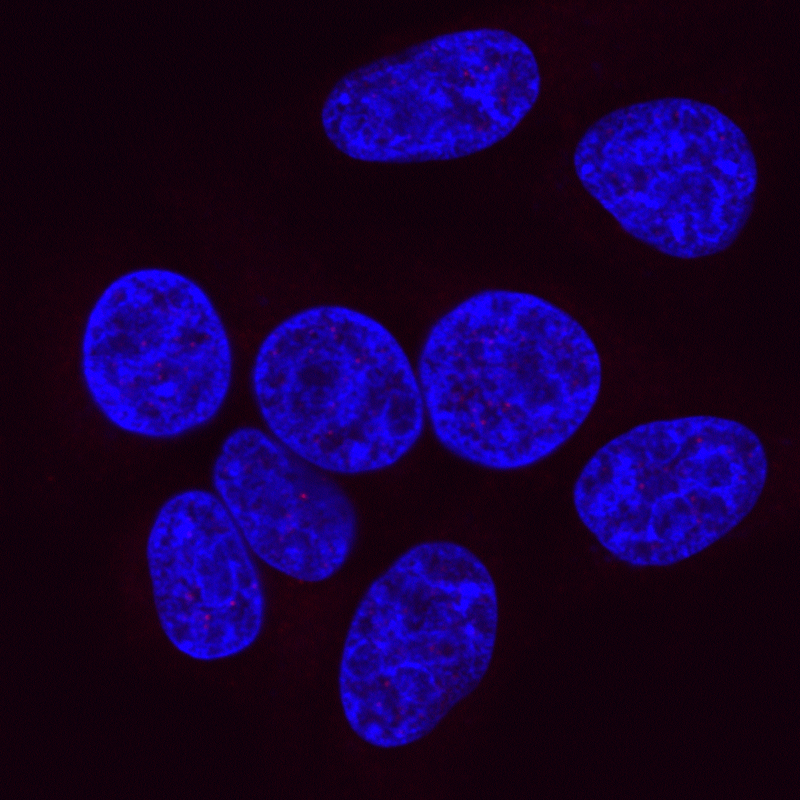

Supplement: Supplementary file 33 [file LSA-2024-02774_SdataF5.2.zip › SourceDataForFigure5B/CoV4803GL.png]

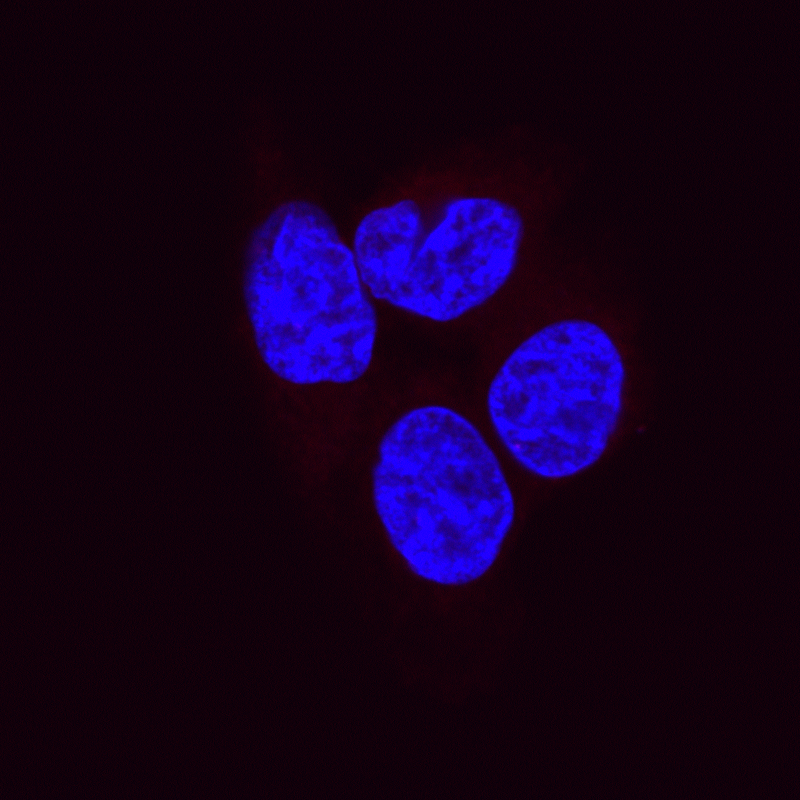

Supplement: Supplementary file 33 [file LSA-2024-02774_SdataF5.2.zip › SourceDataForFigure5B/CoV59020GL.png]

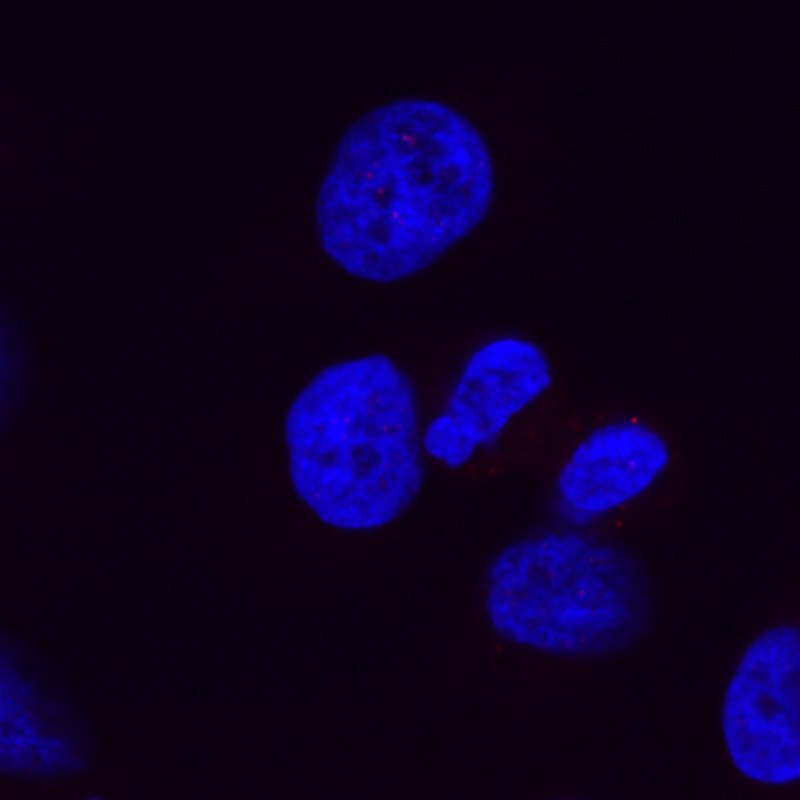

Supplement: Supplementary file 33 [file LSA-2024-02774_SdataF5.2.zip › SourceDataForFigure5B/CoV6100GL.png]

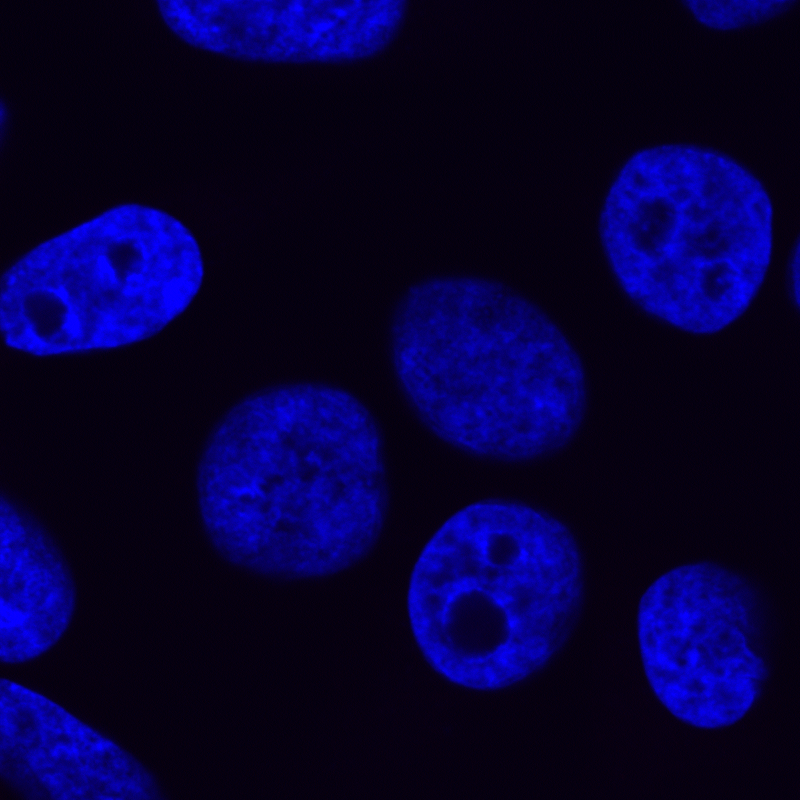

Supplement: Supplementary file 33 [file LSA-2024-02774_SdataF5.2.zip › SourceDataForFigure5B/Negative_control.png]

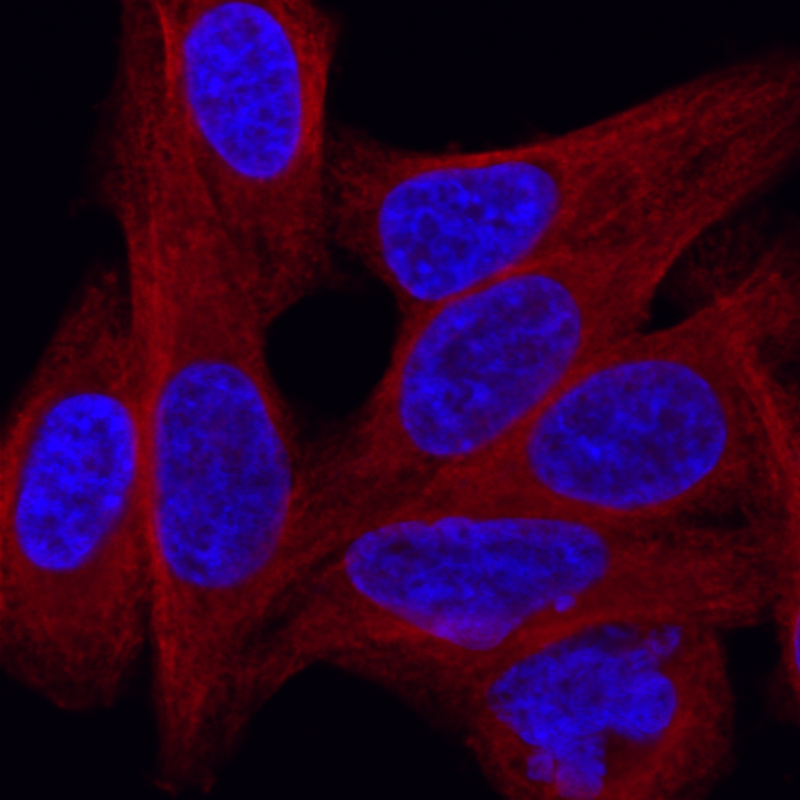

Supplement: Supplementary file 37 [file LSA-2024-02774_SdataF6.4.zip › SourceDataForFigure6E/CoV1810.png]

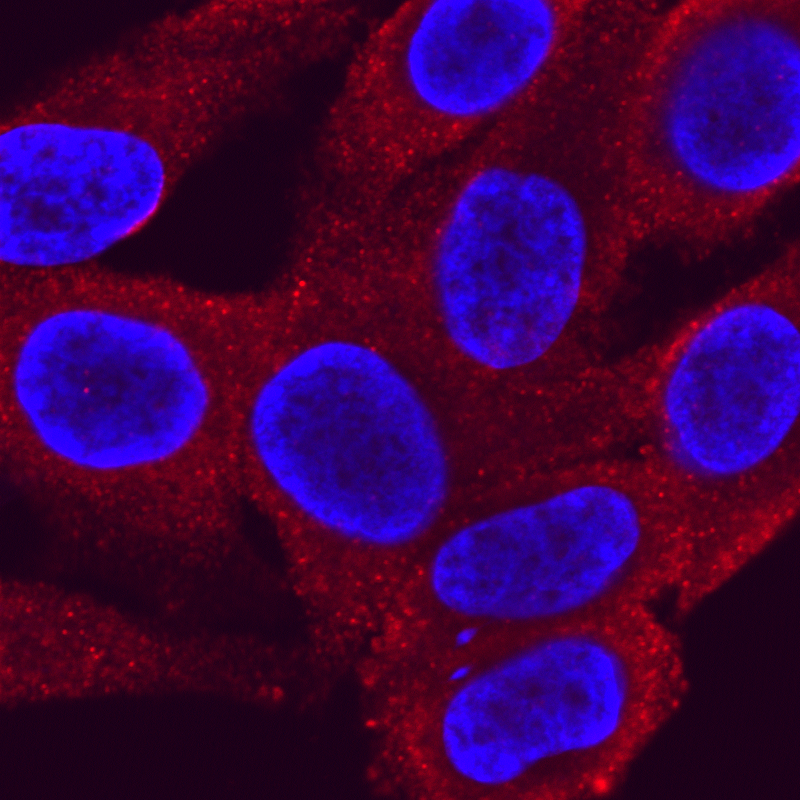

Supplement: Supplementary file 37 [file LSA-2024-02774_SdataF6.4.zip › SourceDataForFigure6E/CoV1827.png]

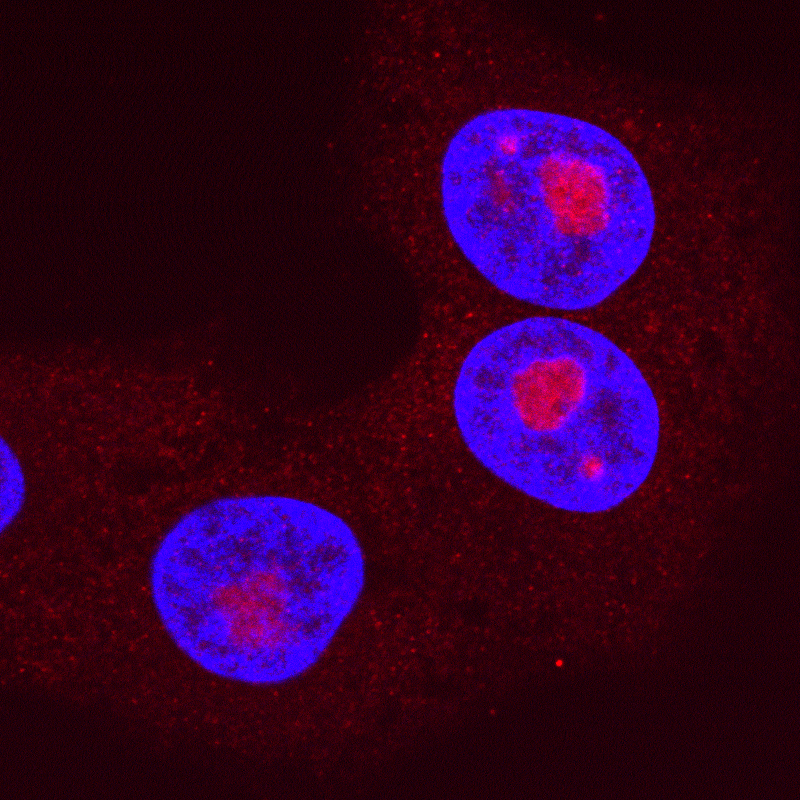

Supplement: Supplementary file 40 [file LSA-2024-02774_SdataF6.7.zip › SourceDataForFigure6J/CoV6100.png]

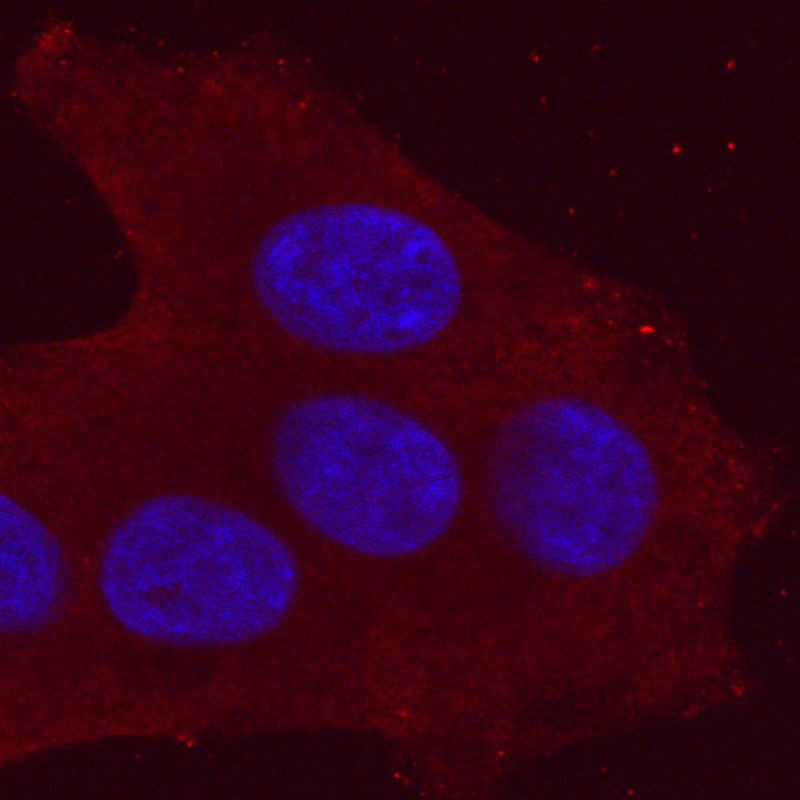

Supplement: Supplementary file 40 [file LSA-2024-02774_SdataF6.7.zip › SourceDataForFigure6J/CoV6111 .png]

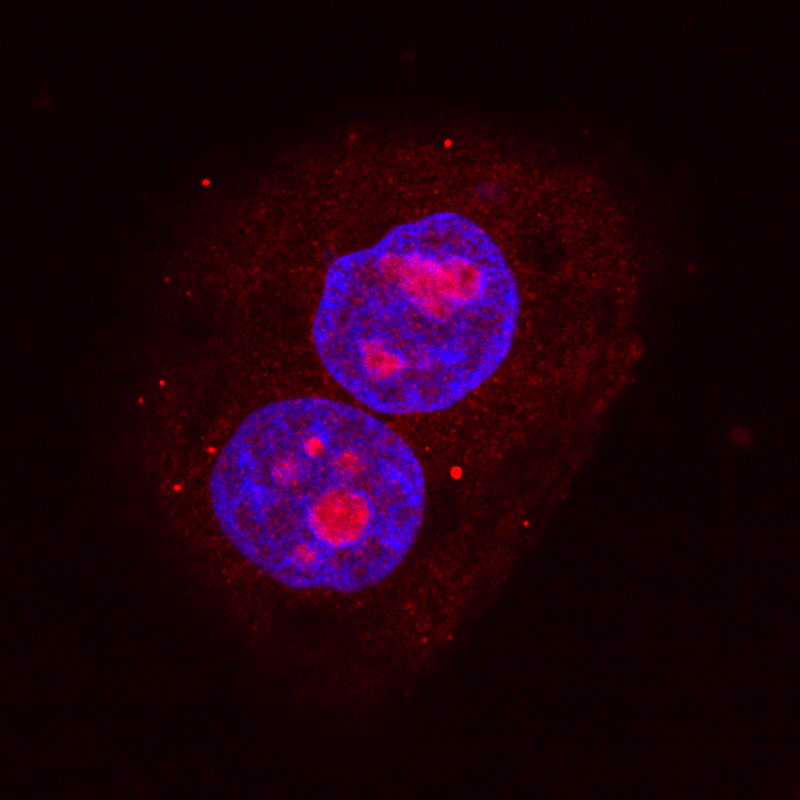

Supplement: Supplementary file 40 [file LSA-2024-02774_SdataF6.7.zip › SourceDataForFigure6J/CoV6114.png]

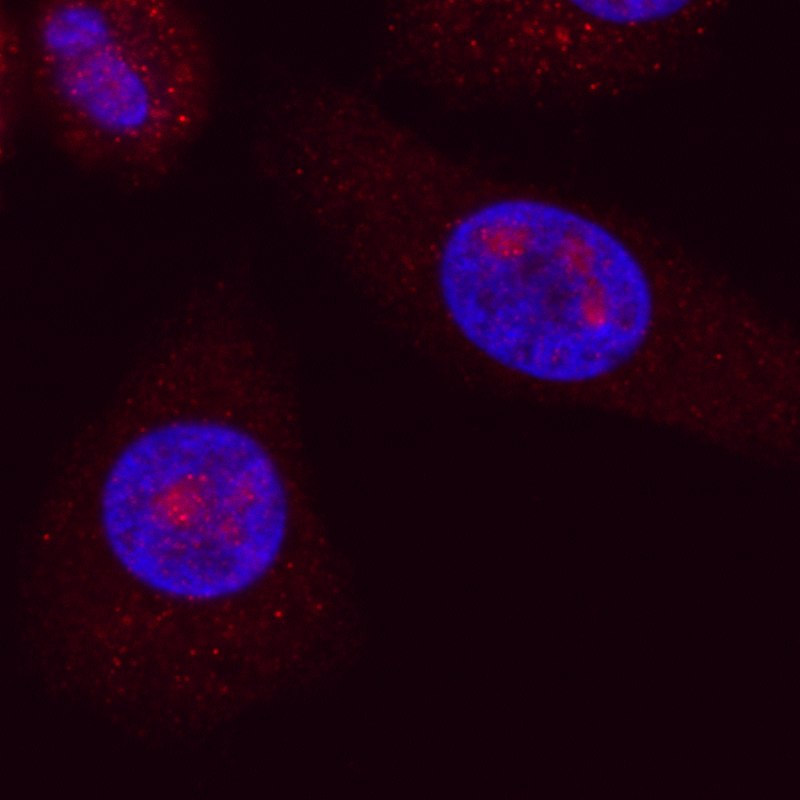

Supplement: Supplementary file 40 [file LSA-2024-02774_SdataF6.7.zip › SourceDataForFigure6J/CoV6139.png]
